# Effectiveness of regionalized systems for stroke and myocardial infarction

**DOI:** 10.1002/brb3.398

**Published:** 2015-09-23

**Authors:** James P. Rhudy, Marie A. Bakitas, Kristiina Hyrkäs, Rita A. Jablonski‐Jaudon, Erica R. Pryor, Henry E. Wang, Anne W. Alexandrov

**Affiliations:** ^1^ School of NursingUniversity of Alabama at BirminghamAlabama; ^2^ Center for Nursing Research and Quality OutcomesMaine Medical CenterBirminghamAlabama; ^3^ Department of Emergency MedicineUniversity of Alabama at BirminghamBirminghamAlabama; ^4^ College of NursingUniversity of Tennessee Health Sciences CenterMemphisTennessee

**Keywords:** Acute ischemic stroke, effectiveness, outcomes, ST‐segment elevation myocardial infarction

## Abstract

**Background:**

Acute ischemic stroke (AIS) and ST‐segment elevation myocardial infarction (STEMI) are ischemic emergencies. Guidelines recommend care delivery within formally regionalized systems of care at designated centers, with bypass of nearby centers of lesser or no designation. We review the evidence of the effectiveness of regionalized systems in AIS and STEMI.

**Methods:**

Literature was searched using terms corresponding to designation of AIS and STEMI systems and from 2010 to the present. Inclusion criteria included report of an outcome on any dependent variable mentioned in the rationale for regionalization in the guidelines and an independent variable comparing care to a non‐ or pre‐regionalized system. Designation was defined in the AIS case as certification by the Joint Commission as either a primary (PSC) or comprehensive (CSC) stroke center. In the STEMI case, the search was conducted linking “regionalization” and “myocardial infarction” or citation as a model system by any American Heart Association statement.

**Results:**

For AIS, 17 publications met these criteria and were selected for review. In the STEMI case, four publications met these criteria; the search was therefore expanded by relaxing the criteria to include any historical or anecdotal comparison to a pre‐ or nonregionalized state. The final yield was nine papers from six systems.

**Conclusion:**

Although regionalized care results in enhanced process and reduced unadjusted rates of disparity in access and adverse outcomes, these differences tend to become nonsignificant when adjusted for delayed presentation and hospital arrival by means other than emergency medical services. The benefits of regionalized care occur along with a temporal trend of improvement due to uptake of quality initiatives and guideline recommendations by all systems regardless of designation. Further research is justified with a randomized registry or cluster randomized design to support or refute recommendations that regionalization should be the standard of care.

## Introduction

Acute ischemic stroke (AIS) and ST‐segment elevation myocardial infarction (STEMI) are common emergency conditions. Each year in the United States, approximately 87% of the 795,000 new or recurrent strokes are AIS and about a third of the 915,000 new or recurrent acute coronary syndromes (ACS) are STEMI. About three per thousand persons per year are affected, resulting in about 22% of all U.S. deaths and costing the U.S. economy about a quarter of a trillion dollars (Go et al. 2013). Emergency care providers have proposed treatment of these conditions within regionalized systems which (1) designate the level of disease‐specific capability of each hospital in a region and (2) establish procedures for emergency medical services (EMS) to bypass facilities of no or lesser designation (Institute of Medicine, 2010). The rationale is to properly match patient need with hospital capability instead of defaulting to the nearest hospital which may lack the required capability.

**Figure 1:**
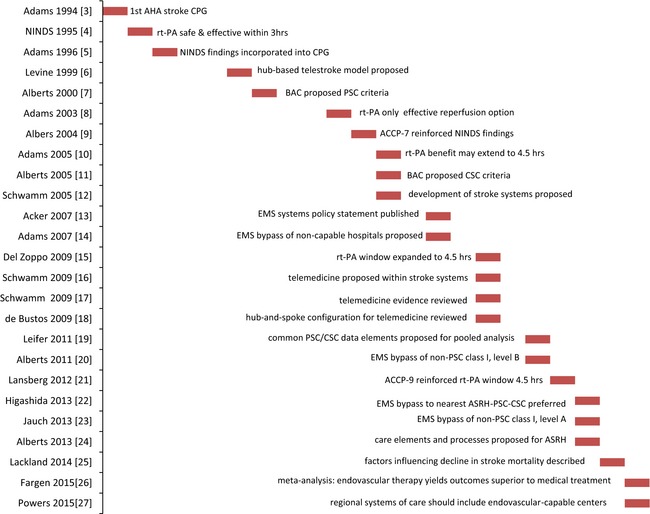
History of guideline statements in acute ischemic stroke.

The history of clinical practice guideline statements for AIS is depicted in Figure 1 (Adams et al. 1994, 1996, 2003, 2005, 2007; The National Institute of Neurological Disorders and Stroke rt‐PA Stoke Study Group, 1995; Levine and Gorman 1999; Alberts et al. 2000, 2005, 2011, 2013; Albers et al. 2004; Schwamm et al. 2005; Acker et al. 2007; de Bustos et al. 2009; Del Zoppo et al. 2009; Schwamm et al., 2009a,b; Leifer et al. 2011; Lansberg et al. 2012; Higashida et al. 2013; Jauch et al. 2013; Lackland et al. 2014; Fargen et al. 2015; Powers et al. 2015). Soon after the determination that recombinant tissue plasminogen activator (rt‐PA) was the first effective therapy (The National Institute of Neurological Disorders and Stroke rt‐PA Stoke Study Group, 1995), stakeholders in stroke care convened the Brain Attack Coalition (BAC) which recommended criteria for primary stroke centers (PSC) (Alberts et al. 2000) to provide the care that most patients would need, and comprehensive stroke centers (CSC) (Alberts et al. 2005) to provide more advanced stroke care. In 2003, the Joint Commission (JC) began to certify PSC based on these criteria. The development of stroke systems of care (Schwamm et al. 2005), EMS systems of prehospital care (Acker et al. 2007), and protocols for EMS bypass of hospitals not capable of stroke care was recommended (Adams et al. 2007). In 2011, the EMS bypass recommendation was graded class I, level B (Alberts et al. 2011); it was upgraded to its current class I, level A in 2013 (Jauch et al. 2013), indicating adequate support by evidence from studies with a randomized design. The BAC has proposed criteria for acute stroke‐ready hospitals to evaluate and triage patients to PSC and CSC as appropriate. Components of a modern stroke care system have been described, including public education outreach focused on symptom recognition and EMS use by patients, prompt recognition and proper triage by EMS personnel, appropriate transport protocols within and between hospitals to match the care with the patient's need, certification of stroke centers to improve outcomes, and implementation of telemedicine systems to support stroke care in rural areas. Endovascular therapy has recently been associated with superior outcomes compared to medical therapy (Fargen et al. 2015). The current focused update of the AIS guideline of the American Heart Association/American Stroke Association recommends that regional systems of care include endovascular‐capable centers for care to be delivered to selected patients (Powers et al. 2015).

**Figure 2:**
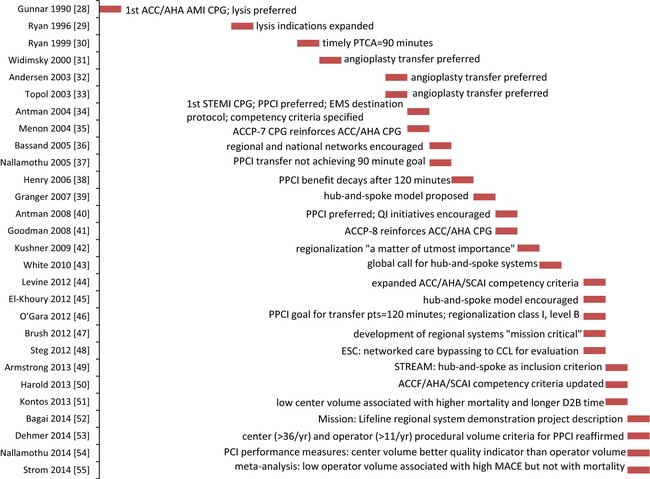
History of guideline statements in ST‐segment elevation myocardial infarction.

The history of guideline statements for AMI and STEMI is depicted in Figure 2 (Gunnar et al. 1990; Ryan et al. 1996, 1999; Widimsky et al. 2000; Andersen et al. 2003; Topol and Kereiakes 2003; Antman et al. 2004, 2008; Menon et al. 2004; Bassand et al. 2005; Nallamothu et al. 2005, 2014; Henry et al. 2006; Granger et al. 2007; Goodman et al. 2008; Kushner et al. 2009; White 2010; Brush 2012; El Khoury et al. 2012; Levine et al. 2012; O'Gara et al. 2012; Steg et al. 2012; Armstrong et al. 2013; Harold et al. 2013; Kontos et al. 2013; Bagai et al. 2014; Dehmer et al. 2014; Strom et al. 2014). The first guideline published in 1990 recommended thrombolytic therapy for patients with AMI symptoms and a pattern of ST‐segment elevations (Gunnar et al. 1990). In 1996, thrombolysis indications were expanded and angioplasty was proposed as an alternative only if it could be implemented in a timely manner by experienced personnel at a high‐volume facility (Ryan et al. 1996). In 1999, “timely” was defined as within 90 min of hospital arrival (Ryan et al. 1999). Randomized trials in the Czech Republic in 2000 (Widimsky et al. 2000) and Denmark in 2003 (Andersen et al. 2003) demonstrated superior outcomes with transfer for angioplasty compared to thrombolysis. In 2004, the first guideline specifically addressing STEMI referred to primary percutaneous coronary intervention (PPCI) indicating that techniques such as stent implantation could result in superior outcomes compared to simple balloon angioplasty and recommended PPCI as the preferred reperfusion strategy (Antman et al. 2004). This guideline recommended development of EMS destination protocols to PPCI‐capable centers and specified procedural volume criteria as a proxy for competence. Later guidelines and scientific papers have urged the provision of care within regionalized systems as “a matter of utmost importance” (Kushner et al. 2009) and “mission critical” (Brush 2012). The “Mission: Lifeline” regionalized system demonstration project is underway; comparisons of outcomes to accomplishments of nonparticipating systems have not yet been published (Bagai et al. 2014). The current guideline recommends regionalization of STEMI care as class I, level B, indicating a lack of support in studies with a randomized design (O'Gara et al. 2012). Volume criteria for centers specify at least 36 primary procedures per year (Harold et al. 2013); this threshold has been associated with lower mortality and shorter door‐to‐balloon times (Kontos et al. 2013). Center volume has been described as a better proxy for competence than operator volume (Nallamothu et al. 2014). Low operator volume has been associated with higher rates of major adverse cardiac events but not increased mortality (Strom et al. 2014).

The purpose of this article is to provide a narrative review of the effectiveness of regionalized systems in AIS and STEMI sufficient to identify the gap between the adequacy of evidence in the AIS case and the deficiencies of the evidence in the STEMI case.

## Methods

### Search strategy

Literature searches were conducted with terms corresponding to designation of AIS and STEMI centers. Effectiveness was defined as outcome on a dependent variable corresponding to any rationale for regionalization specified in the references cited in Figures 1 and 2. In the AIS case, the terms “primary stroke center” OR “comprehensive stroke center” were searched in PubMed and Scopus, limited to the English language and publication within the trailing 5 years. Evidence regarding the effectiveness of JC‐certified systems is provided in Table 1. In the STEMI case, since there is currently no standard means of designation agreed upon by national stakeholders, the terms “regionalization” AND “myocardial infarction” were searched in PubMed and Scopus and limited as above. The yield was augmented by additional searches in two stages. First, literature was searched for papers describing the effectiveness of model systems as described in any reference cited in Figure 2 (North Carolina; Minneapolis, Minnesota; Boston, Massachusetts; France; Denmark; the Czech Republic; and Vienna, Austria). Finally, literature was searched for any paper with a title including the phrase “A Report from the American Heart Association Mission: Lifeline Program.” Criteria for a pre‐ or nondesignated comparison group were relaxed to include any anecdotal or statistical comparison. Papers without any pre‐ or nondesignated comparison were excluded.

**Table 1:** Clinical effectiveness of regionalization in acute ischemic stroke (AIS)

Reference	Design/*Setting*/Sample/Variables	Principal findings	Critique/*Interpretation*
Lichtman et al. (2011a)	Retrospective review of Medicare data; 37,439 patients with SAH or ICH in 2006 DV: in‐hospital and 30‐day all‐cause mortality and 30‐day all‐cause readmission IV: receipt of care at certified (*n* = 11,013) versus noncertified centers (*n* = 26,456)	Unadjusted and risk‐adjusted in‐hospital and 30‐day mortality was lower at certified hospitals for SAH and ICH subgroups No difference in readmission by subgroup or status on certification	Nonreceipt or termination of aggressive care in elderly patients may have confounded analysis There was insufficient data to adjust for stroke severity *JC‐certified status is associated with decreased early mortality but not decreased readmission rates in Medicare population with SAH and ICH*
Lichtman et al. (2011b)	Retrospective review of Medicare data; *n* = 310,381 patients with ischemic stroke in 2006 DV: 30‐day RSMR and 30‐day RSRR IV: hospital status on certification (certified *n* = 63,455, noncertified *n* = 246,926)	Receipt of care at certified hospitals was associated with lower in‐hospital mortality, shorter LOS, and lower rates of disposition to skilled nursing facility or extended care facility Readmission rates were similar by hospital status on certification	Nonreceipt or termination of aggressive care in elderly patients may have confounded analysis There were insufficient data to adjust for stroke severity *JC‐certified status is associated with decreased early mortality, shorter LOS, and favorable disposition but not with lower readmission rates in Medicare population with ischemic stroke*
Panezai et al. (2014)	Retrospective analysis of *New Jersey* Acute Stroke Registry (NJASR) database; *n* = 36,892 patients with acute stroke at 53 PSC (60%) and 12 CSC (40%) during 2010–2011 DV: adherence to JC core measures and median door‐to‐thrombolysis time IV: level of designation	Door‐to‐thrombolysis time was shorter at CSC versus PSC for both 3‐h and 4.5‐h windows Proportion of eligible patients receiving thrombolysis was 19.5% at CSC versus 9.6% at PSC for the 3‐h window and 22.4% versus 11% for the 4.5‐h window	State CSC designation became available in 2007; state PSC designation was implemented gradually beginning in 2010 during data collection, which could have confounded analysis *State CSC designation is associated with core measure adherence*
Bhattacharya et al. (2013)	Chart review at five JC‐certified (*n* = 302) and five noncertified (*n* = 300) hospitals in *Michigan* DV: performance of core process measures IV: hospital certification status; patient demographics	Overall rate of rt‐PA use was low and did not differ by status on certification AA race was associated with delayed presentation Self‐presentation versus EMS was associated with nonreceipt of rt‐PA Core measure compliance was better at certified hospitals, especially for Caucasian patients All disparities were present only, or more pronounced, at noncertified hospitals All disparities n.s. adjusted for delay	The retrospective design and chart review methodology did not allow for detailed investigation of delayed presentation or nonuse of EMS *JC certification mitigates disparity in access to care but does not increase rt‐PA use adjusted for delayed presentation and nonuse of EMS*
Rajamani et al. (2013)	Chart review at five JC‐certified (*n* = 302) and five noncertified (*n* = 300) hospitals in *Michigan* DV: rt‐PA receipt, various process measures, disposition at discharge IV: hospital certification status	3.8% of AIS presenters received rt‐PA (no difference by certification) 48.2% of eligible presenters received rt‐PA at certified versus 8.8% at noncertified centers Certified status was associated with receipt of core performance measures AA race and atrial fibrillation were associated with mortality	Analysis likely confounded by attainment of certification by two of the noncertified hospitals shortly after study's end *JC‐certified status is associated with higher rt‐PA use for eligible patients but not with improved outcomes*
Mullen et al. (2013b)	Retrospective cohort study of *Nationwide Inpatient Sample*;* n* = 323,228 from 2004 to 2009 DV: rt‐PA receipt IV: patient demographics, comorbidities, and APR‐DRG mortality risk; hospital characteristics teaching status, urban versus rural location, region, AIS volume, PSC designation	Overall rt‐PA use low, at 3.1% (6.7% certified, 2.2% noncertified) and doubled during study period Certified status associated with greater rt‐PA use in all analyses Overall proportion receiving rt‐PA doubled during the study period	Analysis likely confounded by implementation of 4.5 h window and participation in telestroke networks and QI initiatives by noncertified centers *JC‐Certified status is associated with increased unadjusted and adjusted rates of rt‐PA use*
Prabhakaran et al. (2012)	Retrospective analysis of *Illinois* Hospital Association CompData; *n* = 119,539 patients during 2003–2009 DV: rt‐PA receipt IV: receipt of care at non‐PSC or at PSC >1 year before, ≤1 year before, ≤1 year after, or > 1 year after certification	The overall proportion of rt‐PA receipt was 5.7% and did not increase by year At non‐PSC, rt‐PA receipt was 1.2% at baseline and increased by year At PSC, rt‐PA receipt increased by designation	“Drip and ship” presenters could not be distinguished from front‐door presenters which might understate rt‐PA use by non‐PSC Confounder: State PSC legislation passed during data collection *PSC status is associated with increased rt‐PA use which begins before certification is achieved*
Prabhakaran et al. (2013)	Six‐month pre/post analysis of effect of 2009 *Illinois* Primary Stroke Center Act with EMS bypass of non‐PSC in Chicago DV: rt‐PA use in those clinically eligible, EMS use, process times, complications of rt‐PA, mortality IV: pre‐ (*n* = 1075) versus postimplementation (*n* = 1172)	Postimplementation unadjusted rt‐PA use was 10.1% versus 3.8% preimplementation Postimplementation phase was associated with increased EMS use, EMS prenotification of stroke center, timely presentation within 3 h and 4.5 h, and decreased process time; performance sustained in 2nd postimplementation year No difference in D2N time, mortality, or rate of intracerebral hemorrhage	Analysis likely confounded by hospital characteristics including years of PSC experience and implementation of 4.5 h window, as well as indirect effect of public education There were no data from non‐PSC in Illinois or from centers achieving PSC status after data collection began *EMS bypass of noncertified centers is associated with increased rt‐PA use*
McKinney et al. (2011)	Retrospective review of *New Jersey* Myocardial Infarction Data Acquisition System (MIDAS) database; *n* = 134,441 patients with cerebral infarction during 1996–2007 DV: 90‐day mortality IV: receipt of care at state‐designated CSC (23.4%), PSC (51.5%), or noncertified center (25.1%); weekend versus weekday presentation	Overall mortality was greater for weekend versus weekday presenters Receipt of thrombolysis increased 4× versus baseline with 2003 JC designation and 10× versus baseline with 2007 state designation 90‐day mortality decreased versus baseline Receipt of thrombolysis was greatest at CSC followed by PSC and noncertified centers	Present designations were attributed to centers for period before designation began State designation was available only during final year of data collection *Weekend effect of increased mortality is mitigated by CSC care but not by PSC care* *State but not JC designation is associated with decreased 90‐day mortality* *JC or state designation is associated with tPA receipt*
Johnson et al. (2014)	Retrospective analysis of *North Carolina* Stroke Care Collaborative data; *n* = 29,654 cases at 47 hospitals during 2005–2010 DV: compliance with JC performance measures plus receipt of rt‐PA, defect‐free care, and statin therapy IV: status on JC PSC certification (PSC+, *n* = 14,885 cases at 10 hospitals; preparing for PSC, *n* = 6974 at 8 hospitals; and non‐PSC, *n* = 7795 at 29 hospitals)	PSC and pre‐PSC had higher compliance than non‐PSC Defect‐free care was most common at PSC followed by pre‐ and non‐PSC Substantial improvement was noted in all measures and subgroups PSC continued to improve performance adjusted for patient and hospital characteristics after achieving certification	Database participation is voluntary, introducing selection bias The greatest increases in performance were seen where performance at baseline was low There was an overall secular trend toward improvement *Achievement of, and preparation for, PSC certification is associated with sustained improvement in performance*
Ballard et al. (2012)	Retrospective cohort study of 17 hospitals in *Kaiser Permanente* integrated system; *n* = 26,727 patients during 2005–2008 DV: process time for ED throughput, utilization of imaging tests, outcomes including LOS, disposition, and mortality IV: care pre‐ (*n* = 15,687) versus postcertification (*n* = 11,040)	Postcertification period associated with faster ED throughput for hemorrhagic subgroup and increased use of MRI for ischemic subgroup No difference in disposition, LOS, or mortality by certification	Variability in coding practices prevented analysis of rt‐PA use and adjustment for stroke severity The culture change across the integrated system as PSC certification was achieved by one hospital at a time may have confounded the analysis *Certified status is associated with enhanced process but not increased survival*
Lewis et al. (2011)	Retrospective review of GWTG‐Stroke database; *n* = 94,119 stroke patients with atrial fibrillation or flutter eligible for anticoagulation during 2003–2010 DV: anticoagulant use IV: demographic factors, hospital characteristics, year of study	Receipt of anticoagulation was >90% by year 2 and remained high Hospital characteristics (more beds, location in the West, academic status, JC certification) were associated with adherence Demographic factors (AA or Hispanic race, diabetes, peripheral vascular disease, carotid stenosis) associated with nonadherence	Data were self‐reported by hospitals without external validation NIHSS not consistently reported; adjustment for stroke severity was not possible *JC‐certified status is associated with improved adherence to anticoagulation performance measure*
Albright et al. (2012)	Review of Specialized Programs of Translational Research in Acute Stroke (SPOTRIAS) database; *n* = 2090 patients at eight CSC during 2002–2009 DV: rt‐PA receipt, in‐hospital mortality, 90‐day functional outcome, disposition, 90‐day mortality IV: weekend versus weekday presentation	There was no significant difference in rt‐PA treatment rates or any other outcome studied between weekend and weekday presenters	Data were collected before formal CSC designation was available, although all centers met CSC criteria Exclusion of patients requiring intra‐arterial therapy limits generalizability *CSC designation mitigates the weekend effect of increased mortality*
Mullen et al. (2013b)	Publication from REGARDS prospective national cohort study oversampling AA and residents of the *U.S. southeastern Stroke Belt* 30,239 individuals with 1000 incident strokes DV: receipt of initial evaluation at PSC versus nondesignated hospital IV: demographic, historical, and clinical variables; Stroke Belt residence and rurality	Factors associated with decreased likelihood of PSC evaluation: Stroke Belt residence, female gender, prior history of stroke Factors associated with increased likelihood of PSC evaluation: male gender, hypertension, income < $20,000	Self‐nomination for certification introduces selection bias Self‐report may be unreliable in a syndrome which could interfere with accurate recall by the patient *JC certification does not mitigate regional disparity in access to care*
Fonarow et al. (2013)	Retrospective analysis of *GWTG‐Stroke* database; *n* = 400,707 ischemic stroke admissions during 2010–2012 DV: Performance Achievement Award (PAA) measures; all‐or‐none defect‐free care; ratio of measures received to measures for which patient was eligible IV: hospital status on PAA and JC certification PAA+/PSC+ *n* = 169,302 PAA+/PSC− *n* = 129,454 PAA−/PSC+ *n* = 26,386 PAA−/PSC− *n* = 75,565	Defect‐free care and unadjusted conformity with measures were greatest at PAA+ hospitals regardless of PSC status, followed by PAA−/PSC+ and PAA−/PSC− hospitals Receipt of rt‐PA was highest at PAA+/PSC+ and lowest at PAA−/PSC− hospitals Adjusted compliance was highest at PAA+/PSC+ hospitals followed by PAA+/PSC−, PAA−/PSC+, PAA−/PSC−	Database participation is voluntary, introducing selection bias Data are self‐reported without external validation NIHSS data were not consistently reported so sensitivity analysis of cases reporting this may introduce selection bias *PAA recognition is a more reliable predictor of performance than PSC certification*
Leira et al. (2012)	Analysis of population coverage by PSC; *n* = 12 PSC in *Iowa* DV: proportion of population residing within a 30‐min distance buffer IV: ungoverned self‐nomination for PSC status versus allocation by maximal coverage model	Self‐selection has resulted in coverage of 37% of Iowa population compared to simulated 47.5% with the same number of PSC allocated by a maximal coverage model	Maximal coverage model disregards other factors which should inform allocation of resources *The current system of self‐nomination for PSC status may not optimally extend coverage to populations*
Lichtman et al. (2009)	Retrospective review of Medicare data for ischemic stroke during 2002; *n* = 366,551 patients DV: in‐hospital and 30‐day all‐cause mortality; 30‐day readmission for all cause, vascular disease, complications IV: receipt of care at hospital which would achieve JC PSC certification during the years 2003–2007 versus noncertified hospitals; patient and hospital characteristics	Unadjusted outcomes for pre‐ versus non‐JC‐certified hospitals are as follows: in‐hospital mortality, 4.7% versus 5.5%; 30‐day mortality, 9.8% versus 11.3%; 30‐day all‐cause readmission, 13.8% versus 14.6%; 30‐day readmission for vascular disease or complications, 7.3% versus 7.9% Risk‐adjusted outcomes were similarly superior at pre‐JC‐certified hospitals	There were insufficient data to study core measure adherence (rt‐PA administration was not a reimbursable code during the study period) or to adjust for stroke severity Results may not generalize to non‐Medicare population *JC‐ and state‐certified status correctly identifies hospitals that have superior outcomes several years before achievement of certification*
Lackland et al. (2014)	American Heart Association and American Stroke Association scientific statement on the factors influencing the decline in stroke mortality	Stroke has declined from the third to the fourth leading cause of death in the U.S.; *the evidence of any impact of JC certification or stroke systems of care on mortality is inconclusive*	The reduction in stroke mortality in both sexes and all age and racial/ethnic groups is valid and multifactorial.

AA, African American; APR‐DRG, all patient refined diagnosis‐related group; D2N, door‐to‐needle; DV, dependent variable; DVT, deep venous thrombosis; ED, emergency department; EMS, emergency medical service; GWTG‐Stroke, Get‐With‐The‐Guidelines for Stroke; ICH, intracerebral hemorrhage; IV, independent variable; JC, the Joint Commission; LOS, length of stay; LTC, long‐term care; n.s., nonsignificant; QI, quality initiative; RSMR, risk‐stratified mortality rate; RSRR, risk‐stratified readmission rate; rt‐PA, recombinant tissue plasminogen activator; SAH, subarachnoid hemorrhage.

## Results

### Acute ischemic stroke

The rationale for regionalized systems in AIS is to optimize clinical and functional outcomes, decrease regional variability, reduce process times, and expand timely access. Lichtman and colleagues demonstrated that JC‐certified status is associated in Medicare patients with decreased early mortality due to subarachnoid or intracerebral hemorrhage (Lichtman et al., 2011a) and with decreased early mortality, shorter hospitalization, and favorable disposition in ischemic stroke (Lichtman et al., 2011b). Neither study demonstrated reduced readmission rates.

A common finding is that certified systems can increase rates of rt‐PA utilization. Panezai et al. (2014) showed that New Jersey CSC designation compared to PSC was associated with shorter process times and twice the proportion of eligible patients receiving thrombolysis. This analysis was limited by implementation of PSC designation after data collection had begun, whereas CSC designation was already established. Bhattacharya et al. (2013) found that JC certification mitigated racial disparities affecting African Americans and improved unadjusted rates of rt‐PA utilization; but, when adjusted for delayed presentation and nonuse of EMS, these findings became nonsignificant. Rajamani et al. (2013) reported that JC‐certified status in Michigan was associated with increased rates of rt‐PA utilization in eligible patients but not favorable disposition or lower mortality. Mullen et al. (2013a) found that JC‐certified status was associated with increased rates of rt‐PA utilization. Their study design did not permit identification of hospitals participating in non‐JC certification programs. Prabhakaran et al. (2012) reported that PSC status is associated with more than doubled rt‐PA utilization that began several years before achievement of certified status and continued to steadily increase; however, there was no difference in mortality or complications (Prabhakaran et al. 2013). The authors acknowledged that not all PSC in the study area were sampled and that this might limit generalizability. McKinney et al. (2011) concluded that state CSC designation in New Jersey, but not PSC designation, mitigated the weekend effect of increased mortality and that state designation, but not JC designation, was associated with decreased 90‐day mortality. State and JC designation were both associated with increased rates of rt‐PA utilization.

Another common finding is that certified status is associated with improved adherence to other core measures. Johnson et al. (2014) demonstrated that JC PSC certification, as well as preparation for certification that would be achieved during the study period, was associated with sustained improvement in core measure adherence in North Carolina. Ballard et al. (2012) reported that PSC designation compared to predesignation performance in the Kaiser Permanente system is associated with reduced process times and improved utilization of timely imaging but not lower mortality, favorable disposition, or shorter hospitalization. Lewis et al. (2011) found that JC‐certified compared to noncertified status was associated with better adherence to the anticoagulation performance measure for patients with atrial fibrillation.

Certified status is associated with mitigation of disparity in access to stroke care. Bhattacharya et al. (2013) demonstrated that JC certification reduced racial disparities in receipt of rt‐PA. Albright et al. (2012) showed that care at hospitals meeting CSC criteria mitigated the so‐called “weekend effect” of increased mortality affecting off‐hours presenters.

Despite the accomplishments reviewed above that have been associated with certified status, there is still room for improvement. Mullen et al. (2013b) reported that 10 years after JC certification became available, residents of the so‐called “stroke belt” of the southeastern U.S. were still underserved. Fonarow et al. (2013) demonstrated that Performance Achievement Award recognition by the American Heart Association (AHA) was a more reliable indicator of performance than JC certification. Leira et al. (2012) concluded that expansion of access by the population of Iowa to care at certified centers would be superior if nomination for certified status were informed by a maximal coverage model compared to the current system of self‐nomination.

Lichtman et al. (2009) compared unadjusted and risk‐adjusted rates of 30‐day mortality and readmission at centers which were early adopters of JC certification to corresponding rates at centers which did not achieve early certification. The authors demonstrated that JC certification identified centers with superior outcomes preceding the availability of the JC certification program by several years. In a recent review of factors associated with overall decreased stroke mortality conducted after 10 years of societal experience with the JC certification program, Lackland et al. (2014) concluded that evidence is inconclusive regarding the effect of certification of stroke systems of care on mortality. Taken as a group, these findings indicate that certified status is associated with superior performance; however, it is more nearly correct that certified status tends to be earned by centers already displaying superior performance and less nearly correct that certified status conveys superior performance capability. Certified status as a factor in superior performance is not well understood when other factors such as delayed presentation, nonuse of EMS, and improved performance by non‐ or precertified centers are also present.

### ST‐segment elevation myocardial infarction

The rationale for interventions specified in the first acute myocardial infarction (AMI) guideline was prevention of mortality (Gunnar et al. 1990), whereas the rationale for interventions specified in the first STEMI guideline was prevention of mortality and clinical and functional benefit from preserved left ventricular function (Antman et al. 2004). Recommendations for regionalization of STEMI care have been justified by expansion of access to reperfusion. Publications from six regionalized systems were found which included (1) at least one dependent variable corresponding to these rationales, (2) care provided by a PPCI‐capable system, and (3) a non‐ or predesignated comparison.

Clemmensen et al. (2013) in Denmark, Kalla et al. (2006) in Austria, Danchin et al. (2008) in France, and Le May et al. (2008) in Ontario, Canada reported decreased mortality with regionalized care. The Denmark report is an anecdotal assertion of an historic low; the other three reports are comparisons with baseline performance. Benedek et al. (2013) reported that implementation of the European Stent for Life quality initiative in Romania was followed by elimination of disparity in mortality affecting those initially presenting to a non‐PPCI‐capable hospital.

Jollis et al. (2012) reported that statewide implementation of the Regional Approach to Cardiovascular Emergencies (RACE) in North Carolina resulted in an historic low proportion of patients clinically eligible but not reperfused. In an earlier report from the RACE system, Jollis et al. (2007) stressed that the study was not designed to examine mortality and that inferences based on their outcomes should be made with great caution. Henry (2012) described the RACE program as a model case of voluntary participation of all hospitals statewide. Glickman et al. (2012) concluded that despite the achievements of this system, mortality was not improved. Glickman et al. (2010) demonstrated that this system reduced all process times for female and elderly patients compared to baseline, significantly mitigating the baseline disparity for female patients, but that the disparity affecting elderly patients persisted.

Forsyth et al. (2012) demonstrated that between 2001 and 2009, the proportion of patients admitted directly to a high‐volume PCI center (then defined as performing >400 PCI procedures per year) in Florida increased from 62.4% at baseline to 89.7% at the study's end. This result was achieved despite the lack of a formal statewide protocol; the interpretation is that superior process can be achieved by “de facto” or self‐organizing systems without formal governance. The system did not eliminate the disparity in admission to high‐volume centers affecting female and elderly patients (Table 2).

**Table 2:** Clinical effectiveness of regionalization in ST‐segment elevation myocardial infarction (STEMI)

Reference	Design/*Setting*/Sample/Variables	Principal findings	Critique/*Interpretation*
Clemmensen et al. (2013)	Report of decade of experience in *Denmark* on the tenth anniversary of DANAMI‐2	Prehospital ECG and helicopter EMS have become available False‐positive CCL activation is low; 30‐day mortality of 5.7% is historic low	Mortality comparison is historical *Effectiveness of STEMI care in Denmark cannot be distinguished from a temporal trend*
Kalla et al. (2006)	Report of expansion of regional system in Vienna, *Austria* over the first 2 years (*n* = 1053)	A single‐center system expanded to include four nonacademic centers Field CCL activation by EMS and community hospital fibrinolysis enabled Proportion of eligible patients not reperfused fell from 34% at baseline to 13.4% In‐hospital mortality fell from 16% at baseline to 9.5%	No randomization Mortality comparison to baseline is historical Strength: report of an unselected consecutive series from an entire community Confounder: increased PPCI utilization *Expansion of a regional system expands access to care and may improve mortality*
Danchin et al. (2008)	Prospective study of all AMI in *France* during 2005 (*n* = 1714) DV: various clinical outcomes IV: reperfusion strategy	Outcomes for reperfused patients better than for nonreperfused patients but did not differ by reperfusion strategy 5‐day mortality for reperfused patients was 4% compared to 8.63% 10 years prior	Difficult to distinguish cause(s) of a 10‐year trend *Effectiveness of nationally protocolized STEMI care cannot be distinguished from a temporal trend*
Benedek et al. (2013)	Prospective pre/post analysis of effect in *Romania* of ESC Stent for Life QI initiative (*n* = 5899 over 8 years) DV: in‐hospital mortality IV: pre‐ versus postimplementation	In‐hospital mortality trended favorably overall and for all subgroups, especially late presenters Mortality disparity at baseline affecting presenters to territorial (noninterventional) hospitals became nonsignificant by year 6	Many territorial hospital presenters are still underserved *Implementation of a continental QI initiative mitigates disparity in outcome affecting patients presenting to a non‐PPCI‐capable hospital*
Le May et al. (2008)	Report of implementation of field EMS referral to CCL in *Ottawa, Ontario, Canada* during the years 2005–2006 DV: mortality, time to reperfusion, reinfarction, stroke, cardiogenic shock, major bleeding IV: field (*n* = 135) versus community hospital CCL referral (*n* = 209)	Field referral group compared to community hospital referral group had a higher proportion with timely reperfusion but no significant difference in any major clinical outcome Overall mortality 5% compared to 10% at baseline	Confounders: increased PPCI receipt, contraindications to fibrinolysis Mortality comparison is historical *Effectiveness of the field‐to‐CCL protocol cannot be distinguished from a temporal trend*
Jollis et al. (2012)	Report of implementation of RACE system in *North Carolina, USA* (*n* = 6841 over 18 months) DV: in‐hospital mortality, proportion of eligible patients reperfused IV: timely versus delayed reperfusion	In‐hospital mortality 2.2% for timely versus 5.7% for delayed reperfusion Proportion eligible but not reperfused fell to 4%, an historic low	The eligible but not treated proportion is an anecdotal comparison *Effectiveness of the RACE protocol cannot be distinguished from a temporal trend*
Glickman et al. (2012)	Secondary analysis of preimplementation RACE data in *North Carolina* (*n* = 12,415) during 2005–2007 DV: unadjusted 30‐day mortality IV: comparison of RACE outcomes to those achieved by nonparticipating NC hospitals and to national benchmark	No difference in mortality between RACE participating and nonparticipating NC hospitals No difference in mortality between RACE participating hospitals and national benchmark	Analyses were based on claims data; important variables affecting mortality may not have been available *Introduction of the RACE STEMI protocol does not improve mortality compared to nonparticipating hospitals or national benchmark*
Glickman et al. (2010)	Pre/post comparison of effect of implementation of RACE on disparities in *North Carolina*;* n* = 2063 STEMI, 1140 to PCI centers and 923 to non‐PCI DV: various treatment times IV: receipt of care during the year pre‐ or postimplementation	All treatment times improved in elderly and female patients compared to baseline There was reduction in disparity affecting female patients at baseline Disparity affecting elderly patients persisted	Comparison with external regional or national benchmarks was not possible; data were self‐reported without external validation *Implementation of the RACE regionalized system mitigates some but not all disparities in access to reperfusion care*
Forsyth et al. (2012)	Retrospective review of statewide hospital discharge surveillance data in *Florida*;* n* = 135,697 STEMI from 2001 to mid‐2009 DV: proportion admitted to high‐volume PCI center in adherence to then‐current guideline specifying >400 procedures per year IV: year of receipt of care	Proportion admitted to high‐volume centers increased from 62.4% at baseline to 89.7% at study's end This occurred despite the absence of a formal statewide STEMI triage protocol Substantial disparities persisted in admission to high‐volume centers affecting female and elderly patients	No analysis possible by mode of presentation (self versus EMS) Transfer‐in patients from integrated systems could not be reliably distinguished from front‐door patients *“De facto” or self‐organizing systems can perform favorably despite lack of formal governance*

AMI, acute myocardial infarction; CCL, cardiac catheterization laboratory; DANAMI‐2, Danish acute myocardial infarction trial‐2; DV, dependent variable; ECG, electrocardiogram; EMS, emergency medical service; ESC, European Society for Cardiology; IV, independent variable; PPCI, primary percutaneous coronary intervention; QI, quality initiative; RACE, reperfusion of acute myocardial infarction in Carolina emergency departments.

## Discussion

In the AIS case, the independent variable in the studies reviewed is status on an actual program of designation by JC certification and the comparison is to a non‐ or predesignation cohort. In the STEMI case, citation as a model system by a guideline statement or a scientific paper was chosen as proxy for designation and comparisons were less often supported by statistical comparisons. Taken as a group, these findings indicate that from a societal perspective, the goals of regionalization are incompletely realized. Most of the studies reviewed share several limitations.

### Selection bias

Most of the studies are retrospective analyses of databases with voluntary participation. Several of the studies are comparisons of prospectively collected postimplementation data to historical preimplementation data reported without external validation. Where authors acknowledged this limitation, they asserted that audits had deemed the data valid. In the STEMI case, citation as a model system cannot occur unless system capabilities include not only clinical practice but also academic pursuits such as publication; this too can introduce selection bias.

### Delayed and/or self‐presentation

Most designs did not allow data collection regarding the proportion of patients who either delayed presentation past eligibility for reperfusion or presented by means other than EMS. The exception is Bhattacharya et al. (2013) who found that self‐presentation compared to EMS use was associated with nonreceipt of rt‐PA. This is likely an unmeasured confounder in other studies.

### Hospital characteristics

The classification of hospitals as certified often included hospitals meeting criteria for designations which did not exist at the time data were collected. In several studies, the implementation of certification programs during data collection was acknowledged as a limitation along with other hospital characteristics.

### Missing data

In several studies, authors acknowledged that data collection had been extended into the past before relevant data were routinely collected because the relevant variables were not yet defined. For example, it was generally not possible to control analyses for stroke severity because NIH Stroke Scale data had not been routinely collected. Historical comparisons for rt‐PA use were difficult because this therapy has not been consistently represented by reimbursable code; it has been shown that assignment of rt‐PA treatment by allocation of procedure code results in an underrepresentation of actual numbers of patients treated Palazzo et al. (2014); Kleindorfer et al. (2008).

### Secular trend

In the AIS case, the comparison group was either a pre‐ or a nondesignated cohort. Improvement in the comparison group was usually noted along with that which occurred in the certified group. In the STEMI case, the comparison was often a historical reference to baseline data without a statistical test. Authors usually acknowledged that their findings could not reliably be attributed to the effect of certification distinct from a secular trend.

### Self‐nomination

In several of the studies reviewed, the authors acknowledged that JC certification is a process for which hospitals self‐nominate. This may not be a nuisance confounder but rather a requirement for a facility whose stakeholders seek to serve in a regionalized system. From a societal point of view, it makes sense to prefer organizations whose leadership is willing, if not eager, to fulfill this responsibility.

### Achievement

Fonarow et al. (2013) demonstrated that superior achievement in adherence to Get With the Guidelines (GWTG)–Stroke measures, and recognition by the AHA on that basis, is a better predictor of performance than JC certification. This may represent the best of both worlds in that voluntary participation in a quality initiative, followed by superior performance, identifies organizations not only willing but also capable of sustained implementation of best practices.

### Recommendations

There is insufficient evidence from randomized designs to support or refute assertions that regionalization should be the standard for stroke care, although the weight of the observational evidence accumulated over the 10 years since JC certification became available indicates that the program is achieving its rationale. Since there is no standard definition of a regionalized system of STEMI care, it is not surprising that the proxy chosen, citation as a model system, yields even less evidence than in the AIS case.

It is important that conceptual clarity be achieved regarding what is, and what is not, a regionalized system so that stakeholders will know whether further efforts at formal regionalization are justified. There are several viable candidate definitions in the STEMI case. The Mission: Lifeline demonstration project is underway Bagai et al. (2014); when outcomes are published, there will be evidence regarding the effectiveness of systems so designated. The AHA sponsors an Accredited Heart Attack (STEMI) Receiving Center program (American Heart Association 2014); hospitals are designated by demonstrating that specific standards have been met. Since the first STEMI guideline (Antman et al. 2004), procedural volume has been proposed as proxy for evidence of competence (Levine et al. 2012; Kontos et al. 2013; Dehmer et al. 2014). Hospitals reporting volume to any database can be ranked from highest to lowest and an appropriate cutpoint can be selected for designation.

It is likely that there would be a great deal of overlap among hospitals with the above characteristics; in fact, the various candidate definitions for facility designation may be conceptually distinct ways of describing and measuring the same phenomenon. The difficulty in finding or generating evidence to either support or refute the STEMI regionalization recommendation is that there is not one explicit definition of the intended system attributes, but rather there several attractive candidate definitions. The accuracy of any research finding is dependent on the use of explicit definitions of variables (Wunsch et al. 2005). The best solution may be the one observed in Florida by Forsyth et al. (2012), who described what happened when participants are allowed to find their own place in the system. It may be that superior organizational ambition and superior outcomes will go hand in hand.

National registries such as Action‐GWTG (American College of Cardiology, American Heart Association) are already in place enabling comparison of risk‐adjusted mortality between a system and its national benchmark. Lauer and D'Agostino (2013) propose the randomized registry trial as a means of generating high‐quality evidence by taking a random sample from such registries. This design would tend to avoid the limitations of the evidence reviewed above, confirm or refute causal links, and enable the pursuit of big data with small budgets.

Chew and Blows (2009) recommend a cluster randomized clinical trial design with the hospital as the unit of analysis, broad inclusion criteria, and data collection including a prespecified outcome of 30‐day mortality and a means of tracking all the associated costs. A large universe of qualified candidate participating hospitals would be required for meaningful random selection to result in a sample suitable for meaningful random assignment to an experimental regionalized protocol or to usual care. If the effect of a universal single‐payer health insurance program is an independent variable of interest, the universe of hospitals should ideally include many candidate hospitals from Canada and the U.S., for example. The complexity of such a design would be enormous; equipoise regarding the merits of networked versus un‐networked care would be necessary and problematic for investigators and stakeholders at all candidate participating hospitals and both governments.

Until evidence from studies with a randomized design is available, the afferent flow of patients from the countryside to specialized centers should be matched with a brisk efferent flow of cognitive skill, decision support, and continuing medical education for rural providers. Decentralized grids of rural emergency response capability should be established or maintained and staffed with properly trained and equipped multidisciplinary teams so that guideline‐adherent emergency care can begin where the patient lives. Regional systems that are in place should ensure that vigorous public outreach is undertaken or continued with a focus on symptom recognition and prompt EMS use.

## Conclusion

Regionalization of emergency care for AIS and STEMI, defined as designation of hospital capabilities and EMS bypass of facilities with no or lesser designation, has been associated with expanded access to care, mitigation of disparity, and enhanced process. Improvement in outcomes due to regionalized care is difficult to distinguish from that which occurs due to quality initiatives and uptake of best practices by all facilities regardless of designation. Further research should include specific comparisons of outcomes achieved by designated systems to national benchmarks, nonregionalized systems, or pre‐regionalized baseline states.

## Conflict of Interest

None declared.
